# Memory effect of arsenic-induced cellular response and its influences on toxicity of titanium dioxide nanoparticle

**DOI:** 10.1038/s41598-018-36455-4

**Published:** 2019-01-14

**Authors:** Su Liu, Bing Wu, Yue Yu, Zhuoyan Shen

**Affiliations:** 1 State Key Laboratory of Pollution Control and Resource Reuse, School of the Environment, Nanjing University, Nanjing, 210023 P.R. China; 2 Department of Environmental Science, School of Engineering, China Pharmaceutical University, Nanjing, 211198 China

## Abstract

Toxicity of arsenic (As) has been widely characterized. However, few studies focus on whether cell responses induced by As at nontoxic concentration could be inherited and further change cell tolerance to another pollutant. In this study, human A549 and HeLa cells were exposed to As at nontoxic concentrations for 10 or 15 passages, then the cells were recovered in the cell medium without As. At 25th passage, residual As in both type of cells was completely removed through the recovery process. And no abnormity in cell viability was identified in both type of cells between control and As-treated groups. Above results indicated that As exposure-recovery treatment had limited influence on phenotype of the cells. However, gene expression profiles determined by high-throughput sequencing showed that As exposure-recovery treatment induced similar expression modification of genes related to inflammation, oxidative stress and epigenetic modulation in the A549 and HeLa cells after recovery of 10 or 15 passages, indicating that As-induced cellular responses have been partially memorized at transcriptional level. The memory effect might play key roles in increased tolerance of the A549 and HeLa cells to adverse effects (cell viability, intracellular reactive oxygen species (ROS) generation and plasma membrane damage) induced by titanium dioxide nanoparticles (as representative pollutant). This study shed new lights on toxic effects induced by As at nontoxic concentration, which is useful for risk assessment of combined effects of As and other pollutants.

## Introduction

Arsenic (As) as one of toxic pollutant receives great attentions because of its high toxicity. Animal experiment studies and clinical observations indicate that As is associated with many kinds of human cancers and noncancerous diseases^1,2^. Recent studies find that As has a potential ability to induce diabetic effect^3,4^. In the study of diabetic effects, the metabolic memory is receiving attention, which was first found in human umbilical vein endothelial cells that were pre-expressed to high glucose. After the cells were exposed to normal glucose, the glucose-induced overexpression of fibronectin was not readily reversible^5^. Now, it has been widely accepted that diabetic animals and patients can continue to develop inflammation and vascular damage even after achieving glycemic control, confirming the phenomenon of metabolic memory^6–8^. Thus, due to the diabetic effect of As, it is hypothesized that As-induced cell response might also have similar memory effect. However, few studies focus on whether As exposure could induce the memory effect.

Inflammation, oxidative stress and epigenetic modulation might play important roles in the “metabolic memory” of diabetes. These adverse effects could also be induced by As exposure. For example, As exposure could induce reactive oxygen species (ROS) ‐ mediated oxidative damage^9,10^, which further change physiological homeostasis and gene expression equilibrium of cells. Growing evidences indicate that altered gene expressions related to inflammation, oxidative stress and epigenetic changes play major roles in response, regulations and alterations of cellular As toxicity^11,12^. These changed events do not involve changes in nuclear DNA sequences, but alter the gene expression equilibrium^13–17^. However, the cells might adapt beneficially to As exposure, in part, by the altered transcription profiles. If these changes are inherited (or memorized), cellular defense system and response to other stress might be influenced. However, little information is available.

Exposure to chemicals is often episodic and repeated in the real environment, which makes the sequential exposure to chemicals normal^18,19^. Thus, this study was designed to analyze memory effect of As-induced cellular response and its influences on response to other pollutants by sequential exposure. Titanium dioxide nanoparticles (nano-TiO_2_) were chosen as target chemical based on following reasons: (1) Nanomaterials are emerging pollutants that are receiving more and more attentions due to their potential toxicities. They might be sequentially exposed to organisms after traditional pollutants such as metals. Nano-TiO_2_ as one of the most commonly used nanomaterials have high probability of human exposure. For example, a recent study predicted the concentrations of nano-TiO_2_ in surface water to be ~2.17 μg/L^20^. (2) ROS generation and inflammation play important roles in toxicities of nano-TiO_2_^21–23^, which are similar with the mode of action of As toxicity. The similar toxicities can be used to determine the As-induced memory effect on these cellular responses.

In this study, human alveolar basal epithelial cell A549 and human cervical carcinoma cell HeLa were exposed to As at nontoxic concentrations for 10 or 15 passages, then both type of cells were recovered in the culture medium without As. At 25-passage, cell viability and gene expression profiles of both cells were analyzed to determine whether As-induced cellular responses were inherited after 10 or 15 passages of recovery. Finally, the nano-TiO_2_ were exposed to the cells with or without As exposure-recovery treatment, respectively. Cytotoxicities including cell viability, intracellular ROS level and release of lactate dehydrogenase (LDH) were measured and compared. This study provides insights to toxic effects induced by As at nontoxic concentration.

## Materials and Methods

### Cell culture

A549 and HeLa cells were purchased from Shanghai cell bank, Chinese Academy of Sciences. A549 cells were cultured in 1640 medium with 0.3 g/L glutamine. HeLa cells were cultured in Dulbecco’s modified Eagles medium (DMEM) with 0.6 g/L glutamine. Both culture media were supplemented with 10% fetal bovine serum, 3.7 g/L sodium bicarbonate, 0.1 g/L sodium pyruvate, 80 U/mL penicillin and 80 mg/L streptomycin. Cells were seeded in 96-well plates at a density of 15,000 cells/well and incubated at 37 °C and 5% CO_2_.

### Exposure and recovery treatment of As

**Figure 1:**
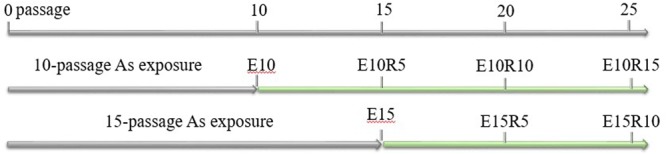
Exposure strategy used in this study. The E10R5 means 10-passage As exposure and 5-passage recovery. Other symbols have the similar meanings.

Exposure concentrations of arsenic trioxide for A549 and HeLa cells were set to 1 and 0.5 μM, respectively, which were the no observed effect concentrations (NOEC) on intracellular ROS generation for both cells (Fig. S1). Arsenic trioxide was supplied by ANPEL laboratory technologies Inc. (Shanghai, China). At the beginning of exposure, each cell line was divided into two groups. One group was exposed to As for 10 passages, and then the cells were recovered in the culture medium without As. Another group was exposed to As for 15 passages and then incubated in the culture medium without As. Expressions on different exposure conditions are shown in Fig. 1. For example, the E10R15 means 10-passage As exposure and 15-passage recovery. Other symbols have the similar meanings.

### Cell viability assay after As exposure-recovery treatment

After As exposure-recovery treatment (the 25^th^ passage of cells), cell viabilities of both cells were determined by cell counting kit (CCK-8) (Dojindo, Japan). Briefly, the cells were incubated with CCK-8 reagent for 1.5 h. Then the absorbance at 450 nm was measured by a microplate reader (Synergy H1, BioTek, USA). Cell viability in treated group was expressed as percentage of viable cells compared to that of control group (cells without As exposure-recovery treatment).

### RNA-seq analyses after As exposure-recovery treatment

The E10R15 and E15R10 treated A549 and HeLa cells were collected to analyze their gene expression profiles. The cells without As exposure-recovery treatment were chosen as the control. Three biological replicates were used for each group. Total RNA was isolated by Takara RNA Kit (Takara Bio. Inc., Japan). The extracted total RNAs were purified by poly-A selection and used for the construction of cDNA libraries. Then the cDNA libraries were applied to perform RNA-sequencing at Novogene Co Ltd. (Beijing, China) on an Illumina HiSeq 4000 platform. Raw reads from sequencing output were processed for the removal of low quality bases according to the following criteria: reads containing adapter, reads containing >10% skipped bases (marked as ‘N’), reads containing 50% bases whose quality score were ≤20. Then the clear reads were mapped to reference human genome using hierarchical indexing for spliced alignment of transcripts (HISAT) software. Fragments per kilobase per millions mapped reads (FPKM) were used to normalize the expression values. Mapping read count data was analyzed by using R DEGseq package to identify the differentially expressed genes (DEGs) with the criteria that the adjusted *p*-value < 0.05. The Metascape program (http://www.metascape.org) was used to identify the Gene Ontology (GO) terms^24,25^, and the biological pathways were identified from the Kyoto Encyclopedia of Genes and Genomes (KEGG, http://www.genome.jp/kegg/).

### Exposure of nano-TiO_2_

The A549 and HeLa cells at 15^th^, 20^th^ and 25^th^ passage in control and As-treated groups were exposed to nano-TiO_2_ for 24 h to perform the cytotoxicity assays. Nano-TiO_2_ (Product No.: XFI02) were obtained from Nanjing XFNANO Materials Tech Co Ltd. (Nanjing, China). Purity and specific surface area of nano-TiO_2_ provided by the manufacture were >99% and 77.37 m^2^/g, respectively. Diameter of nano-TiO_2_ was 10 nm, which was verified by transmission electron microscope (TEM) and scanning electron microscope (SEM) images (Fig. S2).

### Cytotoxicity assay of nano-TiO_2_

Cytotoxicities induced by nano-TiO_2_ were determined by cell viability, generation of intracellular ROS and LDH release. Based on the effective concentrations of nano-TiO_2_ on cell viability from literatures^26,27^, the 0–50 mg/L were chosen as exposure concentrations. Cell viability was determined by using the CCK-8 kit (Dojindo, Japan). Intracellular ROS levels were measured by 2,7-dichlorofluorescin diacetate (DCFH-DA, Invitrogen, USA). LDH releases were performed by LDH assay kit (KeyGEN Biotech, China). Detail information on these assays was shown in the supporting information.

### Measurement of intracellular As and Ti concentration

At 15^th^, 20^th^ and 25^th^ passage, intracellular As concentrations were measured. After exposure to nano-TiO_2_ at 25^th^ passage, intracellular Ti concentrations were also measure. The cells in the 6-well plates were collected, centrifuged and rinsed with PBS. Then, 1 mL of distilled water was added to cell pellet and sonicated for 3 min at a power of 60 W with ice bath. Then the homogenization was divided into 100 μL for measurement of protein concentration and 900 μL for determination of As and Ti concentration. Protein concentration was measured by bicinchoninic acid method followed protocol of manufacturers (Beyotime technology, Nantong, China). Briefly, 20 μL of homogenate was mixed with 200 μL staining fluid. Then their absorbance was measured at 595 nm. Protein concentration was calculated according to the standard curve. For measurement of As and Ti concentrations, after the homogenates were centrifuged at 14000 g for 20 min and filtered with 0.22 μm membrane, the As and Ti concentrations were measured by inductively coupled plasma mass spectrometry (ICP-MS) (PerkinElmer, USA). Finally, the As and Ti concentrations were normalized by the protein concentration.

### Statistical analysis

For all assays, three independent trials were performed. Results are expressed as the means ± standard deviation (SD). Statistical differences were evaluated by one-way ANOVA test with Tukey’s post hoc test, which was performed using GraphPad Prism (version 6.0). A value of *p* < 0.05 was considered as statistically significant.

## Results

### Influence of As treatment on A549 and HeLa cells

**Figure 2:**
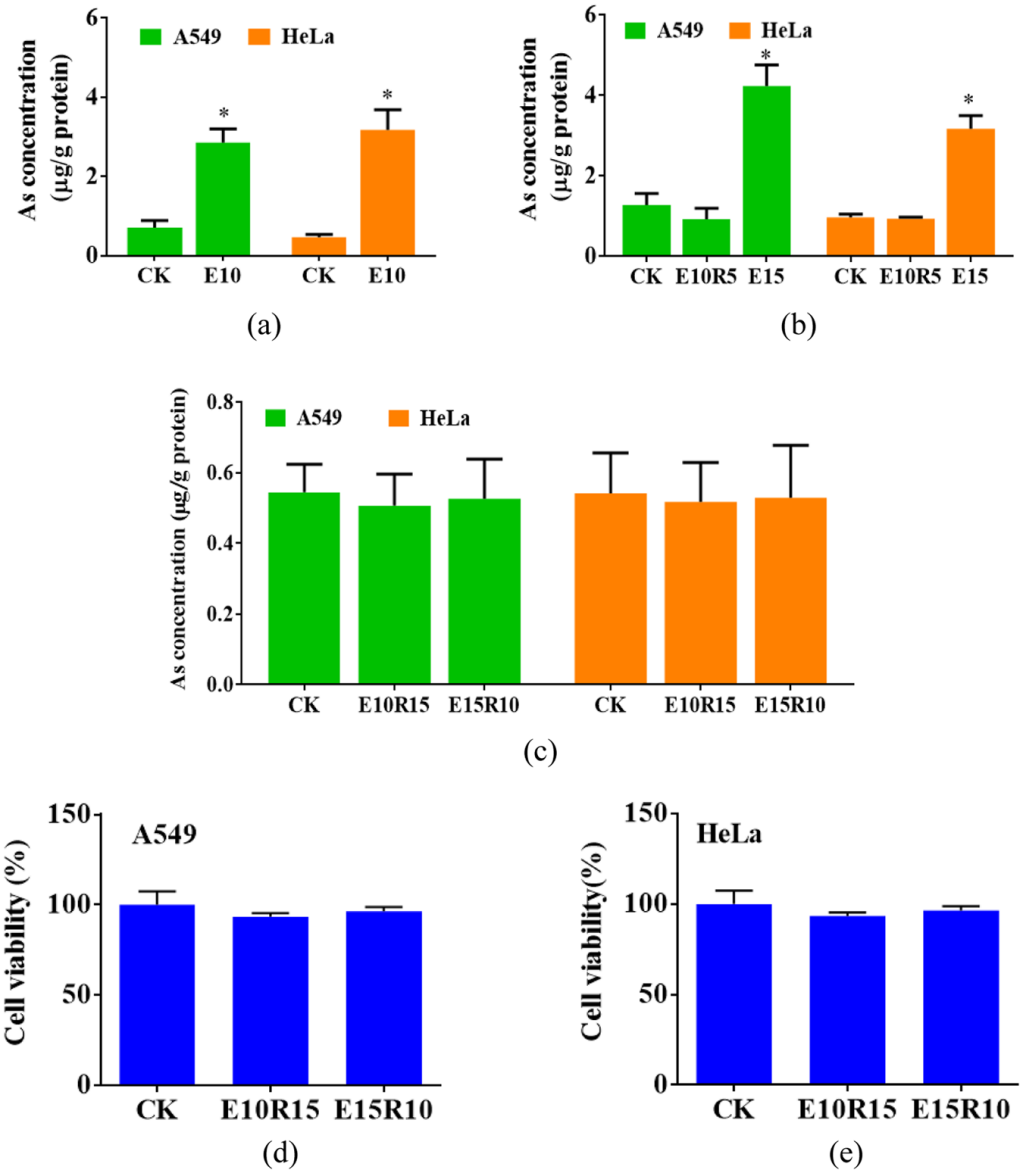
Influence of As exposure-recovery treatments on intracellular As concentration and cell viability of A549 and HeLa cells. (**a**) Intracellular As concentrations at 10^th^ passage. (**b**) Intracellular As concentrations at 15^th^ passage. (**c**) Intracellular As concentrations at 25^th^ passage. (**d**) Cell viability of A549 cells at 25^th^ passage. (**e**) Cell viability of HeLa cell at 25^th^ passage. Results are shown as the mean ± standard deviation. ^✳^Means *p* < 0.05 compared to control cell (CK).

During exposure to As, intracellular As levels in the A549 and HeLa cells significantly increased (Fig. 2a,b). After further cultured in the medium without As, intracellular As concentrations reduced to the level of the control cells (Fig. 2b,c). Moreover, the As exposure-recovery treatments did not change the cell viability in the A549 and HeLa cells among control and treated groups (Fig. 2d,e).

### Gene expression profiles in A549 and HeLa cells

**Figure 3:**
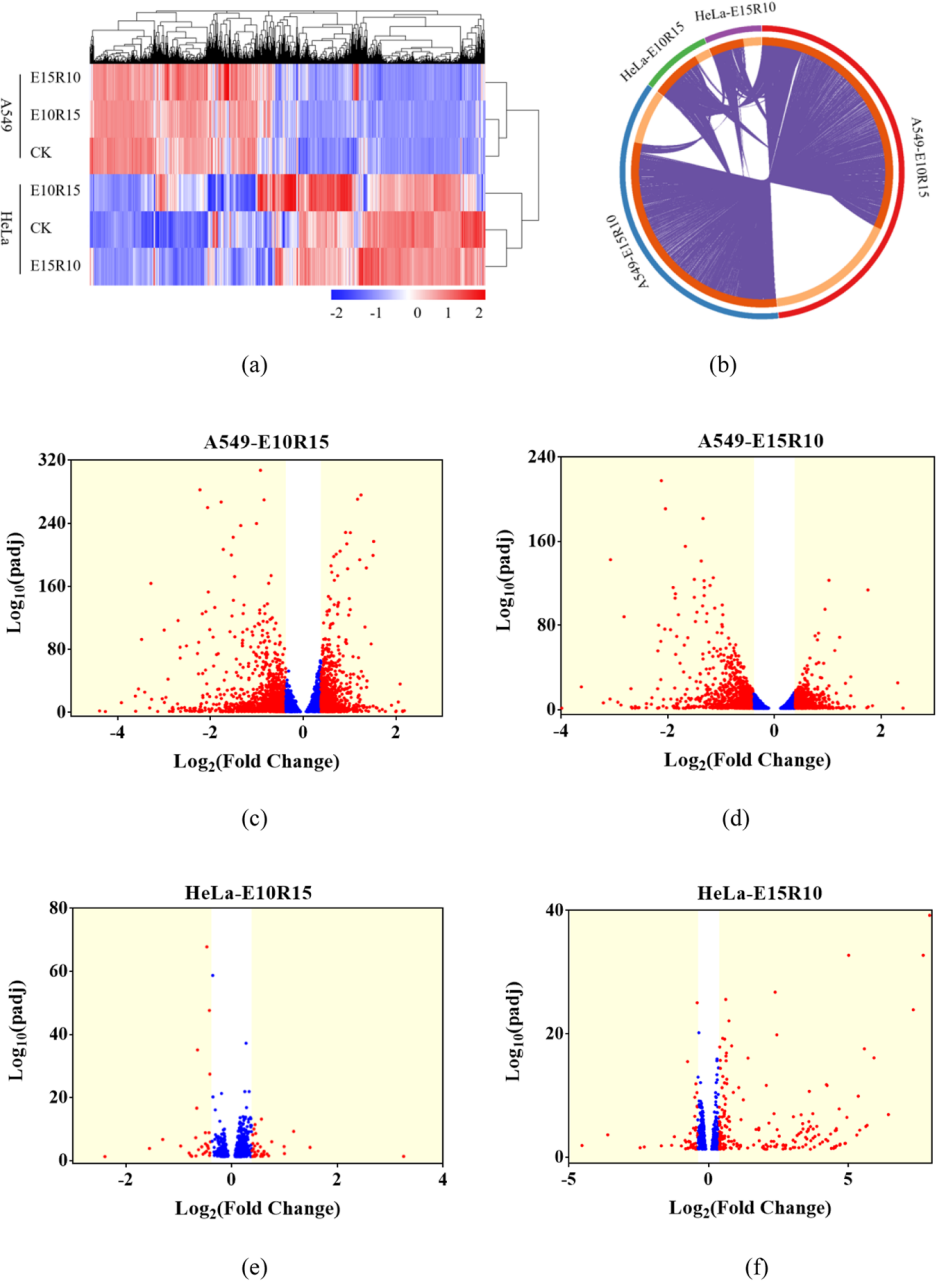
Comparison of DEGs in A549 and HeLa cells after As exposure-recovery treatment. (**a**) Cluster analysis of DEGs. (**b**) Cricos plot of DGEs among four treated groups. (**c**–**f**) Volcano plots between fold change and adjusted p-value of DEGs.

A549 cells with E10R15 and E15R10 treatments posed 6270 and 4820 DEGs compared to control cells, respectively. The HeLa cells with E10R15 and E15R10 treatment had 1061 and 860 DEGs, respectively. Cluster analysis indicated that A549 and HeLa cells had the different gene expression profiles (Fig. 3a). Circos overlap showed that 3795 and 165 DEGs were shared between E10R15 and E15R10 groups for A549 and HeLa cells, respectively (Figs 3b and S3). Additionally, 73 DEGs were shared among the A549 and HeLa cells with E10R15 and E15R10 treatments. Volcano plot showed the relationships between fold change and adjusted *p*-value (Fig. 3c–f). For A549 cell, DEGs with fold change >±1.3 were predominated. However, for HeLa cell, fold changes of most DEGs were lower than ±1.3.

### Altered biological processes based on the shared DEGs

**Figure 4:**
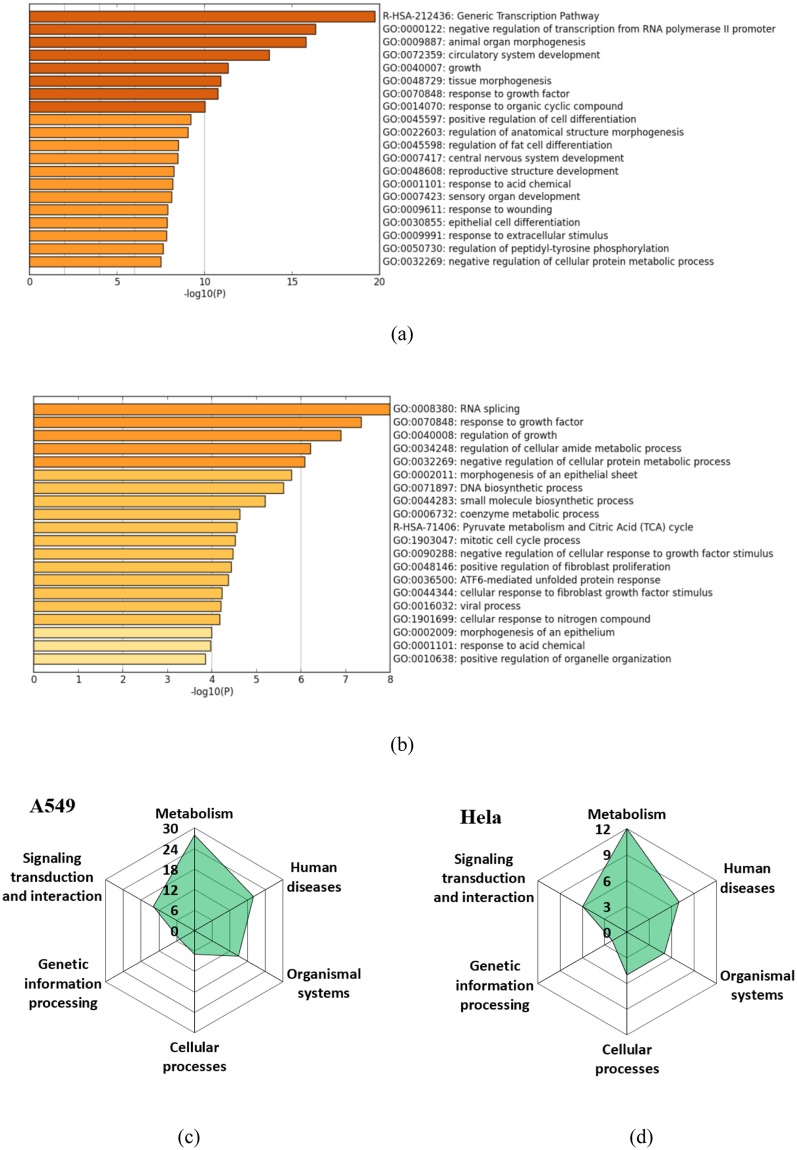
Altered GO terms and KEGG pathways as a consequences of transcriptome changes. (**a** and **b**) Show the top 20 altered GO terms based on the shared DEGs in A549 and HeLa cells, respectively. (**c** and **d**) Show the number of pathway groups in A549 and HeLa cells, respectively, which are displayed as a spider diagram. The axes correspond to the number of significantly altered KEGG pathways belonging to the groups.

In order to characterize the potentially heritable changes in transcriptomic profiles after As exposure-recovery treatment, the shared DEGs in E10R15 and E15R10 treated cells were analyzed based on GO and KEGG databases. Since number of the shared DEGs in treated A549 cells was too high, higher criteria that fold change >±1.5 and *p* < 0.05 were used to identify the DEGs. Then 1404 shared DEGs were identified, which mainly changed negative regulation of transcription from RNA polymerase II promoter, animal organ morphogenesis, circulatory system development, response to growth factor (Fig. 4a). For HeLa cells, 165 shared DEGs mainly changed RNA splicing, response to growth factor and growth, regulation of cellular amide metabolic process (Fig. 4b).

Relationships among the above altered GO terms were further analyzed by network (Fig. S4). For A549 cells, chromatin organization and methylation were clustered into one group. Positive regulation of intracellular signal transduction and negative regulation of cellular protein metabolic process were clustered into another group. For HeLa cells, closer connection among different GO terms than A549 cells was found. Response to growth factor, regulation of cell growth, regulation of cellular protein metabolic process and response to organonitrogen compound had important influence on this network.

The KEGG pathway database provides an alternative resource to map genes against biological processes. Based on the shared DEGs, 94 and 40 altered KEGG pathways for A549 and HeLa cells were identified, respectively. These pathways were assigned to pathway groups provided by the KEGG database. The number of identified pathways in each group was displayed as a spider diagram (Fig. 4c,d)^28^. Most altered KEGG pathways in the A549 and HeLa cells belonged to the ‘metabolism’ and ‘human disease’, followed by ‘signaling transduction and interaction’. Few changes in genetic information processing were found.

The 73 DEGs shared among the A549 and HeLa cells with E10R15 and E15R10 treatments were involved in the response to organophosphorus, coenzyme metabolic process, cell response to growth factor stimulus, RNA splicing (Fig. S5). The KEGG analysis showed that these DEGs changed glycolysis/gluconeogenesis, nitrogen metabolism, urea cycle and metabolism of amino groups, citrate cycle and ribosome.

### Changes in inflammation, oxidative stress and epigenetic modulation

**Figure 5:**
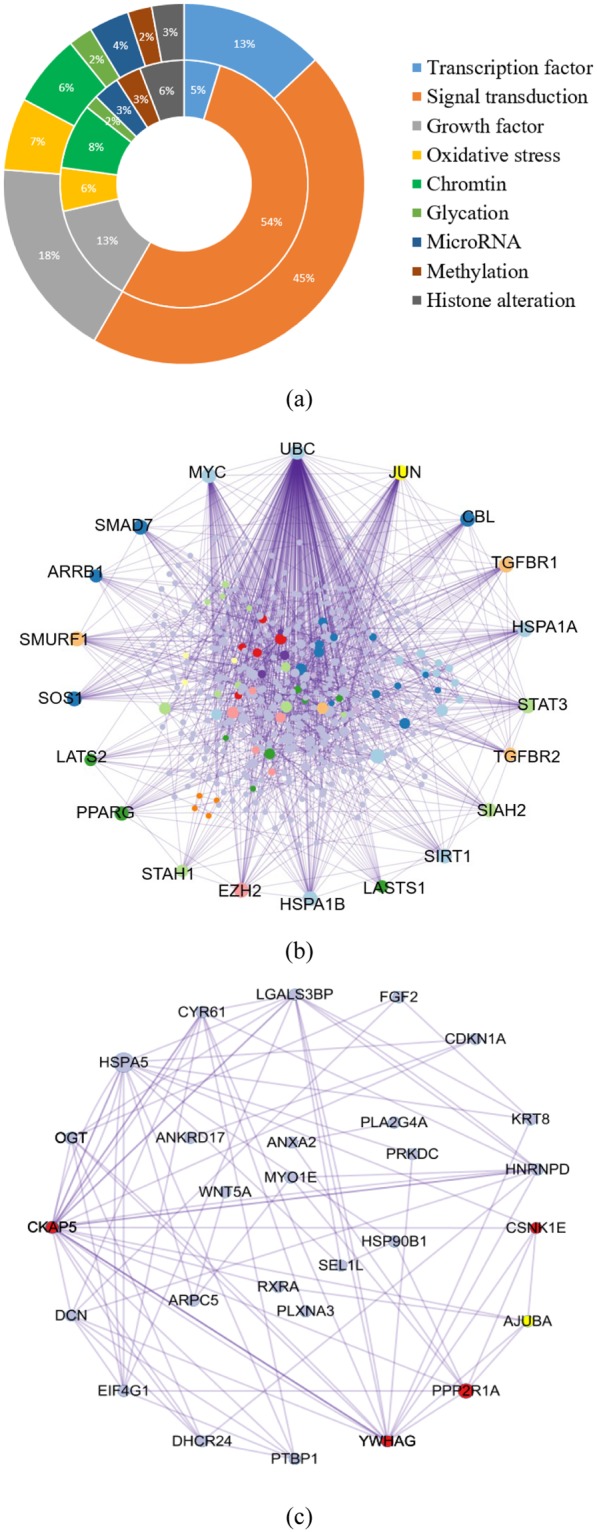
Molecular functions of the DEGs related to inflammation, oxidative stress and epigenetic modulation. (**a**) Percentage of DEGs based on different molecular function. Inner ring shows the percentage for the A549 cell, and outer ring for HeLa cell. (**b**) Network of DEGs in A549 cell based on protein-protein interaction. (**c**) Network of DEGs in HeLa cell based on protein-protein interaction.

The DEGs related to inflammation, oxidative stress and epigenetic modulation were further identified and analyzed, which have been proven to be potential molecular mechanisms of memory effect of diabetic mellitus^29–31^. For A549 cells, a total of 538 DEGs were identified in the E10R15 and E15R10 groups, which accounted for 38.3% of the shared 1404 genes in the both groups. For HeLa cells, 81 DEGs were identified, which accounted for 49.1% of the shared 165 genes in the both groups. Percentages of these obtained DEGs were calculated according to their molecular factions and shown by ring diagram (Fig. 5a). The DEGs related to signal transduction were predominant (53% and 45% for A549 and HeLa cells, respectively), followed by growth factor and transcription factor.

Networks of DEGs involved in inflammation, oxidative stress and epigenetic modulation were constructed based on protein-protein interaction. For A549 cells, the network showed that many genes including *Ubc*, *Myc*, *Jun*, *Stat3*, *Smad7* and *Hspa1b* had very high correlation with other genes in the network (Fig. 5b). The above genes are involved in the signal transduction. For HeLa cells, some genes including *Ywhag*, *Ppp2r1a*, *Fgf2*, *Dcn* and *Hspa5* had high correlation (Fig. 5c). These genes mainly involved in signal transduction. Some other key genes including *Hist1hzbd*, *Stat3*, *Col4a6*, *Sesn2*, *Taf6l*, *Tgfbr2*, *Atxn7* were also found.

### Influence of As treatment on cell response to nano-TiO_2_

**Figure 6:**
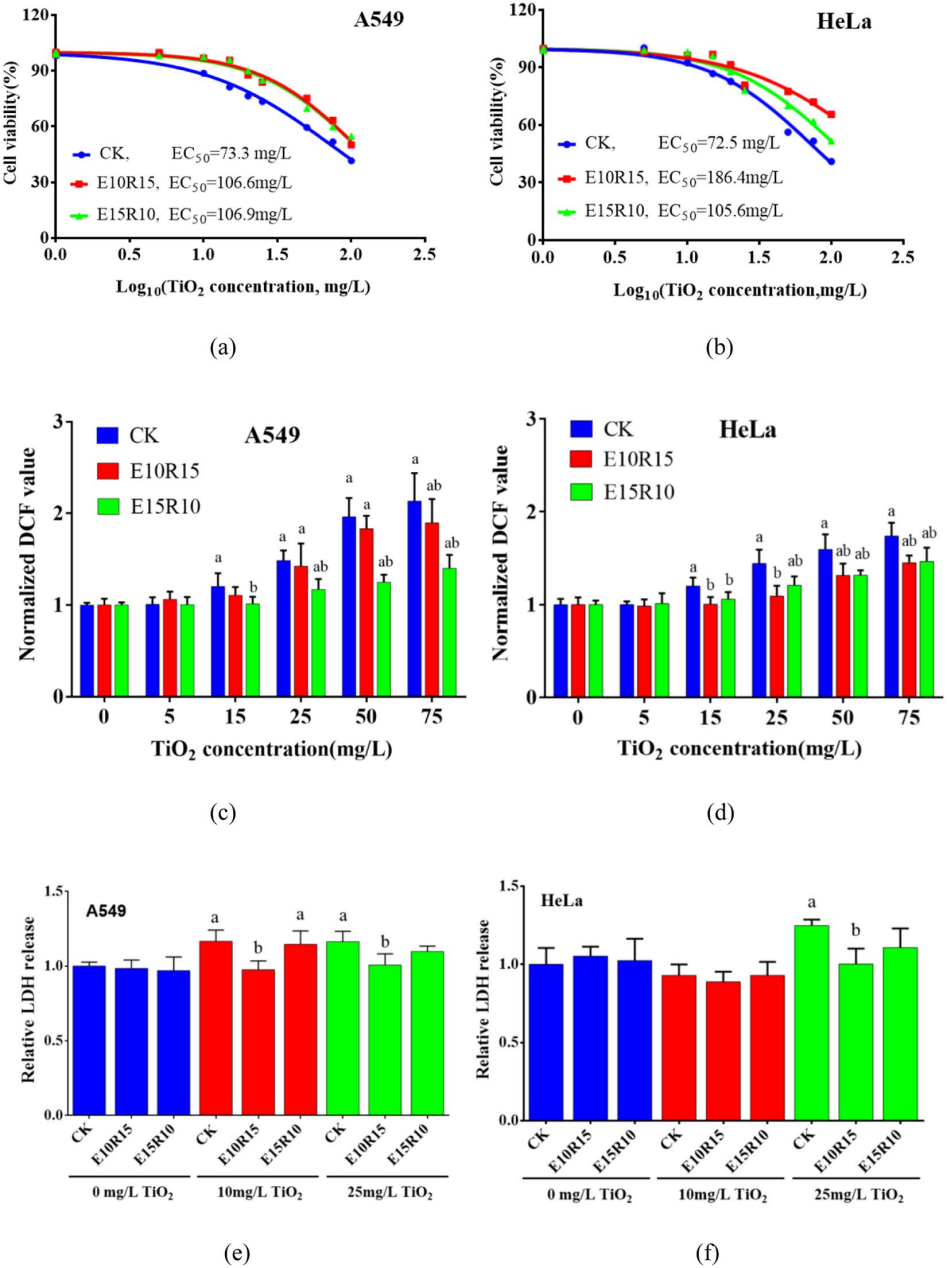
Cytotoxicity of nano-TiO_2_ on A549 and HeLa cells after E10R15 and E15R10 treatments. (**a** and **b**) Show the cell viability. (**c** and **d**) Show intracellular ROS levels. (**e**) and (**f**) Show LDH release. Results of (**c**–**f**) are shown as the mean ± standard deviation. ^a^Means *p* < 0.05 compared to the cells without nano-TiO_2_ exposure. ^b^Means *p* < 0.05 compared to the control cells (CK) after the same As exposure-recovery treatments.

When A549 and HeLa cells after As exposure-recovery treatment were exposed to nano-TiO_2_, no significant difference among intracellular Ti concentrations was found in both cells with or without As treatment (Fig. S6). However, different toxicity responses were found (Fig. 6). Calculated EC_50_ value of nano-TiO_2_ for control A549 cells was 73.3 mg/L, but E10R15 and E15R10 treatments of As increased the calculated EC_50_ values to 106.6 mg/L and 106.9 mg/L, respectively. For HeLa cells, the calculated EC_50_ values after E10R15 and E15R10 treatments of As were 186.4 mg/L and 105.6 mg/L, respectively, which were higher than that of untreated control cells. The increased EC_50_ values for both cells were also found at 15^th^ and 20^th^ passage (Figs S7 and S8).

This study further found that As exposure-recovery treatments decreased generation of intracellular ROS induced by nano-TiO_2_ (Fig. 6c,d). E10R15 treatment of As decreased more ROS generation than E15R10 treatment in A549 cells. However, no significant difference was found in HeLa cells after E10R15 and E15R10 treatments of As. For LDH release, nano-TiO_2_ exposure increased LDH release in the control A549 and HeLa cells (Fig. 6e,f). The As exposure-recovery treatments decreased LDH release in the both cells compared to the control cells, but significant difference was only found in the both cells with E10R15 treatment.

## Discussion

This study analyzed memory effect of arsenic-induced cellular response and its influences on response to other toxic pollutant. The As exposure-recovery treatments did not lead to the intracellular As residual and changes in cell viability (Fig. 2), indicating that As exposure and recovery treatments did not change phenotype of the both cells.

Under normal conditions, cells display a finely tuned gene expression equilibrium. Environmental pollutant exposures could break the equilibrium. Thus, this study further analyzed the influence of As treatment on gene expression profiles. Results showed that number of DEGs in A549 cells was higher than HeLa cells at 25^th^ passage, which might be due to the different exposure concentrations (1 vs 0.5 μM) and cell response profiles between both cells. Since these DEGs were measured after recovery of 10 or 15 passages, thus, the altered gene expression profiles should be partially heritable. Biological meaning of the DEGs were determined based on GO and KEGG pathways. It is interesting that the altered GO terms and KEGG pathways in the A549 and HeLa cells showed high similarity. The altered GO terms in both cells all involved in the transcriptomic process and growth factor (Fig. 4). The altered KEGG pathways mainly referred to metabolism, human disease and signaling transduction and interaction for both cells. The alterations in metabolism and human disease showed As exposure and recovery treatments changed a set of enzymes and other proteins with vital functions^28^, indicating the cell damage and potential irreversible effects. Further, changes in signaling transduction and interaction indicated the disturbance of cellular gene regulations^32,33^. Moreover, the 73 shared DEGs among two cells mainly involved in the gene regulations and signaling pathway, which verified that signaling transduction and functions might play important roles in the memory effect of both cells.

We further analyzed the genes related to inflammation, oxidative stress and epigenetic modulation, which have been proven to be involved in the memory phenomenon in diabetic mellitus^30,31^. DEGs related to these molecular functions accounted for high percentage of the shared DEGs in both A549 and HeLa cells, which strongly indicated that gene regulations followed an overall biological pattern and was not just a random event. These DEGs might play important roles in re-establishing the gene expression equilibrium in both cells. Thus, we deduce that the cells were excited by As exposure on the anti-inflammation, anti-oxidative stress and epigenetic modulation, which could be partially inherited. Current literatures showed that As exposure could disturb pro/antioxidant balance and stimulate defense mechanisms that counter stress by scavenging free radicals and repairing damaged cellular biomolecules. These responses are attempted by changing various pathways such as MAPK signaling pathway (including gene *Fgf2*, *Myc* and *Jun*) and TGF-beta signaling pathway (including gene *Dcn*)^34–37^. The MAPKs are a family of serine/threonine protein kinases that can be involved in the regulation of the synthesis of inflammation mediators at the transcription and translation levels. TGF-beta signaling involved in the regulation of proliferation, apoptosis and inflammation of many cells can activate a number of Smad-independent signaling pathways, including MAPK, PI3-K/Akt, p38, and JNK, which are all involved in the inflammation and oxidative stress pathways. Moreover, after As exposure and recovery treatments, some other genes and pathways (besides signaling pathways) related to anti-inflammation and anti-oxidative stress and epigenetic modulation were also found (Fig. 6). Thus, it might be deduced that these altered pathways might be partially inherited after recovery of 15 passages, which might induce the memory effect of As-induced cellular responses.

In order to confirm whether the changes in gene expression profiles might influence response of both cells to toxic pollutants. This study chose nano-TiO_2_ as representative chemical. Results suggested As exposure-recovery treatments increased tolerance of A549 and HeLa cells to adverse effects (cell viability, intracellular ROS generation and plasma membrane damage) of nano-TiO_2_ (Figs 6 and S7, S8). Moreover, the calculated EC_50_ values of nano-TiO_2_ to each type of cells were similar at the 15^th^, 20^th^ and 25^th^ passage, confirming the memory effect of toxic tolerance to nano-TiO_2_.

There are three potential ways that cells after As exposure-recovery treatments could obtain the ability of tolerating nano-TiO_2_: (1) decreased cellular uptake of nano-TiO_2_; (2) gene mutations induced by As exposure^38,39^; (3) inheritable changes in gene expression profiles^40,41^. This study found that As exposure-recovery treatments did not change cellular uptake of nano-TiO_2_ by A549 and HeLa cells (Fig. S6), suggesting that nano-TiO_2_ uptake was not the reason of increased cell tolerance to nano-TiO_2_. A great amount of evidences showed environmental arsenicals and their methylated metabolites have the capacity to induce genome destabilizing and epigenetic effects in cells and *in vivo*, but have weaknesses at inducing mutations at low doses (conditions of high cell survival like this study)^10^. Thus, gene mutations induced by As exposure might be not the reason of increased cell tolerance to nano-TiO_2_. Previous literatures showed that nano-TiO_2_ exposure could induce oxidative stress, inflammatory response and signaling pathways^42–44^, which were also involved in the memory effect on gene expression profiles induced by As in this study. Thus, the inheritable changes in gene expression profiles might be the main reason of increased cell tolerance to nano-TiO_2_. It should be noted that current data just provides primary explanations on the toxicity tolerance, which are far from being conclusive and more researches need to be performed in future study.

In conclusions, As exposure and recovery treatments did not change phenotype of A549 and HeLa cells, but induced the inheritable modification of expression profiles of genes related to inflammation, oxidative stress and epigenetic modulation, indicating As exposure could induce memory effects on gene expression profiles. The memory effect might play key roles in increased tolerance of A549 and HeLa cells to nano-TiO_2_. This study shed new lights on adverse effects induced by As at nontoxic concentration, which is useful for risk assessment of combined effects of As and other pollutants.

## Electronic supplementary material

Supplementary Information
