# Supplementary material for: Memory effect of arsenic-induced cellular response and its influences on toxicity of titanium dioxide nanoparticle
Source: Sci Rep. 2019 Jan 14;9:107. doi: 10.1038/s41598-018-36455-4 (PMC6331635; doi:10.1038/s41598-018-36455-4)
Supplement: Supplementary file 1 — Supplementary Information [file 41598_2018_36455_MOESM1_ESM.pdf]

Supporting information for

Memory effect of arsenic-induced cellular response and its influences on toxicity of titanium dioxide nanoparticle

Su Liu[1,2], Bing Wu[1]*, Yue Yu[1], Zhuoyan Shen[1]

[1] State Key Laboratory of Pollution Control and Resource Reuse, School of the Environment, Nanjing University, Nanjing, 210023, P.R. China

[2] Department of Environmental Science, School of Engineering, China Pharmaceutical University, Nanjing, 211198, China

*** Corresponding author:**

Tel: +86-25-89680720

E-mail: bwu@nju.edu.cn

Postal address: NO. 163 Xianlin Avenue, Nanjing, 210023, P.R. China

Cell viability assay after nano-TiO_2

After 24 h exposure of nano-TiO_2, cells were rinsed and incubated for 1.5 h with CCK-8 reagent. The absorbance at 450 nm was measured by a microplate reader (Synergy H1, BioTek, USA). Cell viability in treated group was expressed as percentage of viable cells compared to that of control group.

Intracellular ROS assay after nano-TiO_2

Intracellular ROS levels in A549 and HeLa cells were measured by 2,7-dichlorofluorescin diacetate (DCFH-DA, Invitrogen, USA). After 24 h exposure to nano-TiO_2 (0-25 mg/L), 10 μM DCFH-DA was added to cells and incubated for 25 min. Then 2.5 mg/L Hoechst 33342 probe (Invitrogen, USA) was added and incubated for 15 min to complement the cell loss caused by nano-TiO_2 exposure and normalize the fluorescence value of DCF. Fluorescence values of DCF and Hoechst 33342 were measured by a microplate reader (Synergy H1, BioTek, USA). The excitation/emission wavelengths for DCF and Hoechst 33342 were 485/530nm and 350/460nm, respectively.

LDH release assay after nano-TiO_2

The LDH release was performed by LDH assay kit (KeyGEN Biotech, China). Cells were seeded in the 6-well plates and exposed to nano-TiO_2 for 24 h. Then LDH in culture medium was measured. First, the cell culture medium was centrifuged at 13000 rpm for 10 min to remove the nano-TiO_2. Then the experiment was conducted according to the manufacture's introduction. The absorbance of medium at 440 nm was measured by a microplate reader (Synergy H1, BioTek, USA). The LDH release in treated group was presented as the percentage of control group.

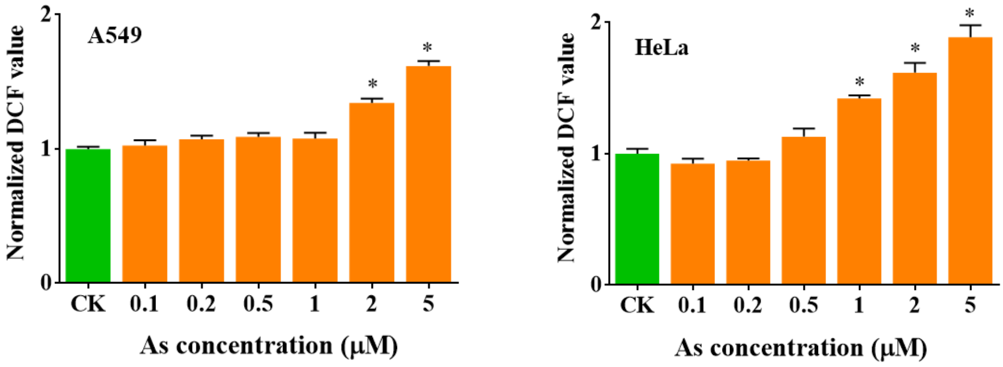

Figure S1 Intracellular ROS levels in A549 and HeLa cells induced by 24h exposure to arsenic. Results are shown as the mean ± standard deviation. * means $p < 0.05$ compared to control cell (CK).

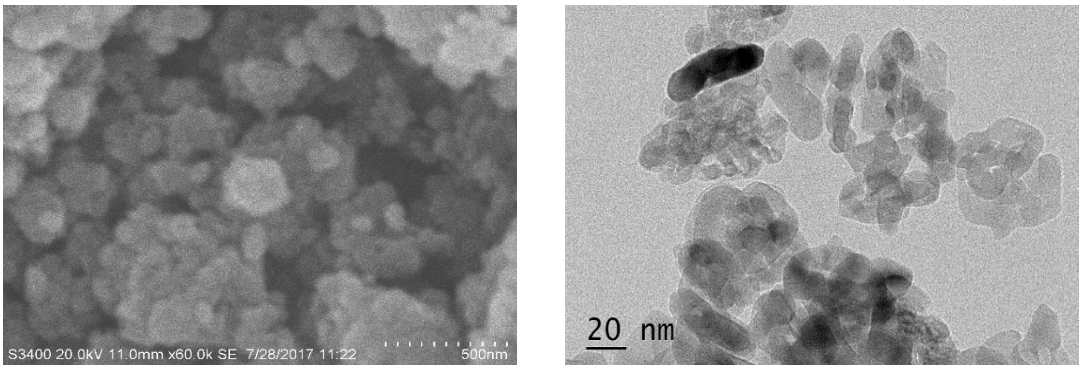

(a) (b)

Figure S2 SEM (a) and TEM (b) images of nano-TiO_2 used in this study.

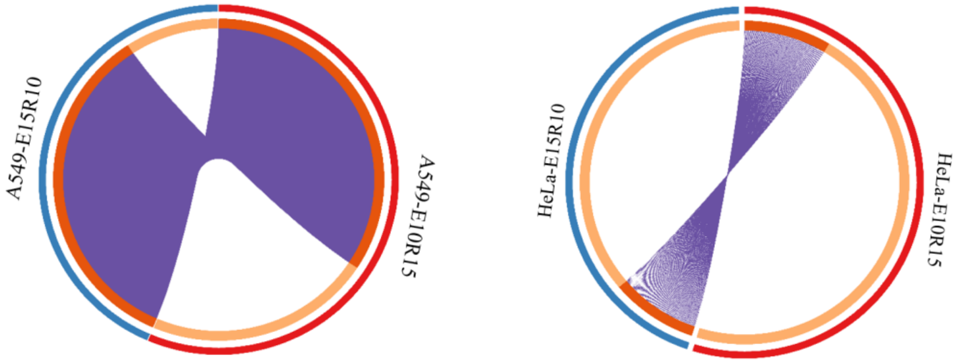

(a) (b)

Figure S3 Cricos plots of differentially expressed genes shared in treated (a) A549 and (b) HeLa cells.

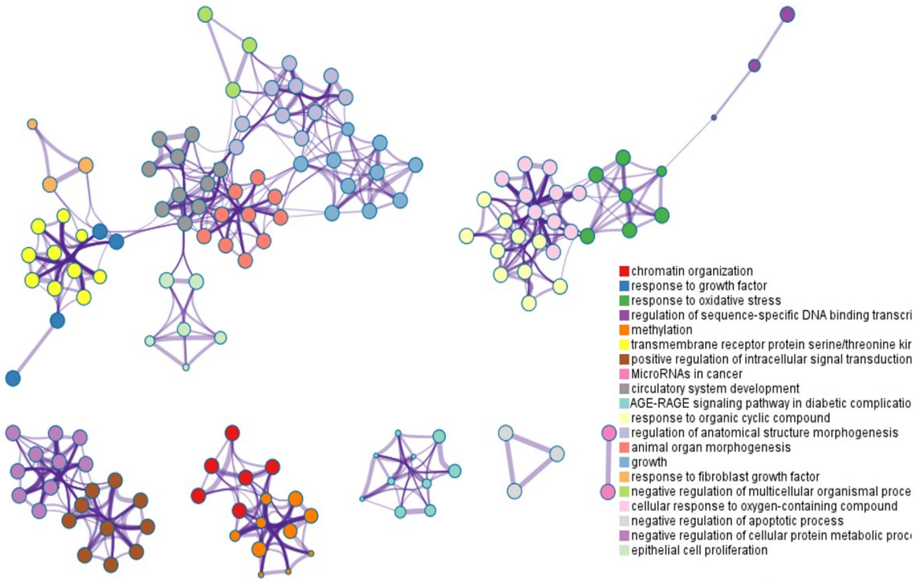

(a)

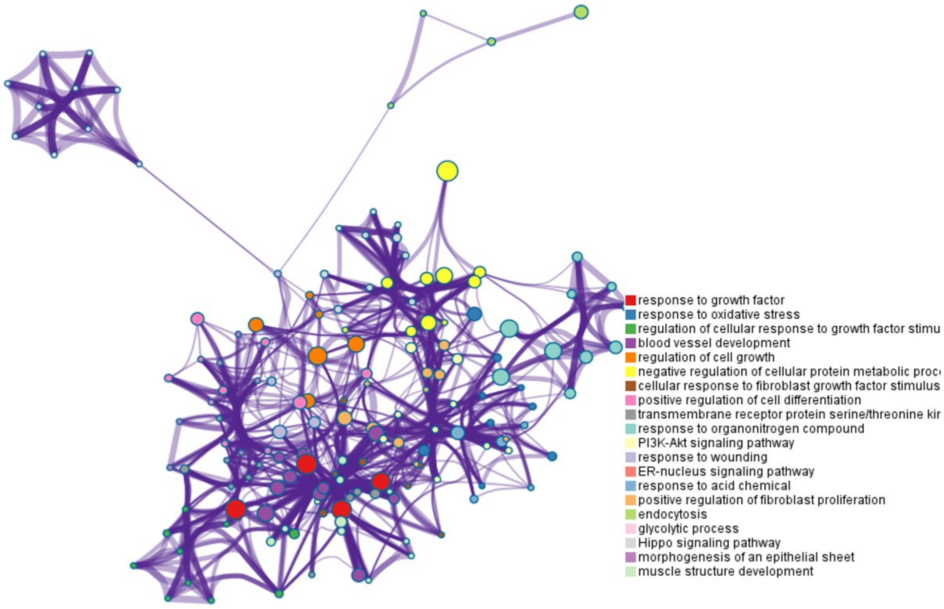

(b)

Figure S4 Network of of representative GO terms in A549 (a) and HeLa (b) cells. The networks were obtained from the Metascpe (http://www.metascape.org). Each term is represented by a circle node, where its size is proportional to the number of input genes, and its color represent its cluster identity (i.e., the same color belong to the same cluster). Terms with a similarity score > 0.3 are linked by an edge (the thickness of the edge represents the similarity score). One term from each cluster is selected to have its term description shown as label.

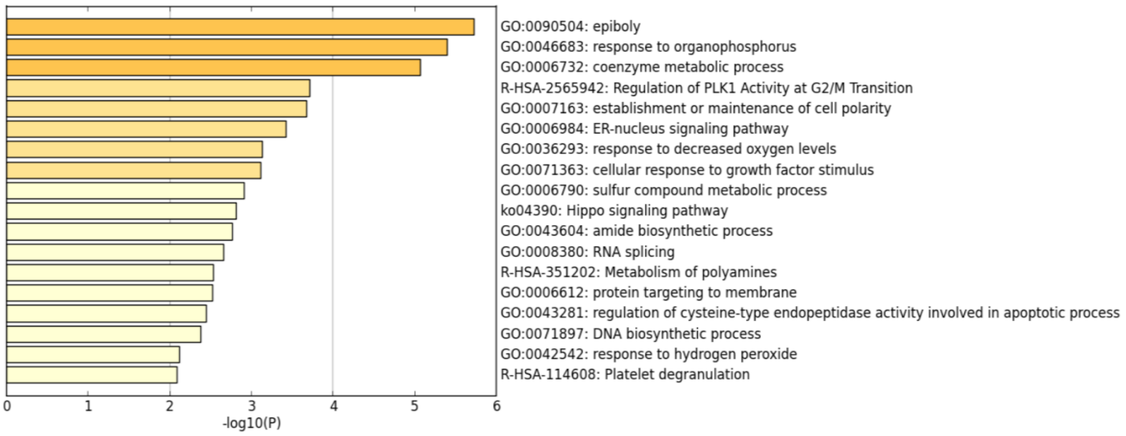

Figure S5 Altered GO terms induced by the 73 differential expressed genes shared among the four treated groups.

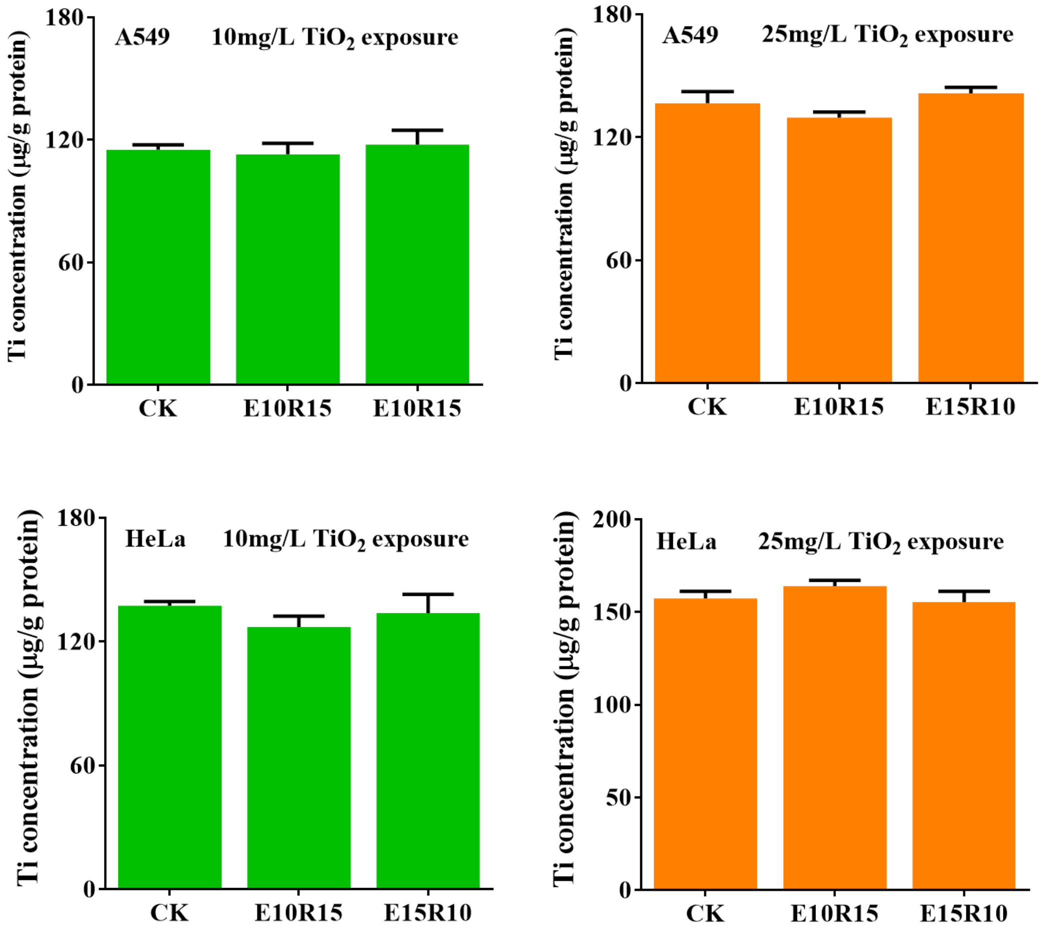

Figure S6 Intracellular titanium (Ti) concentrations in A549 and HeLa cells with E10R15 and E15R10 treatments after 24 h nano-TiO_2 exposure. Results are shown as the mean ± standard deviation. CK means the untreated control cells.

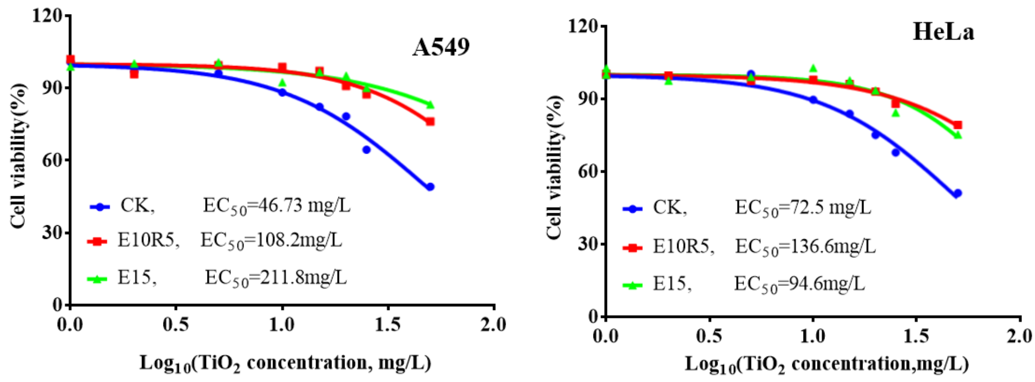

Figure S7 Cell viability of A549 and HeLa cells at 15th passage induced by 24 h exposure to nano-TiO_2.

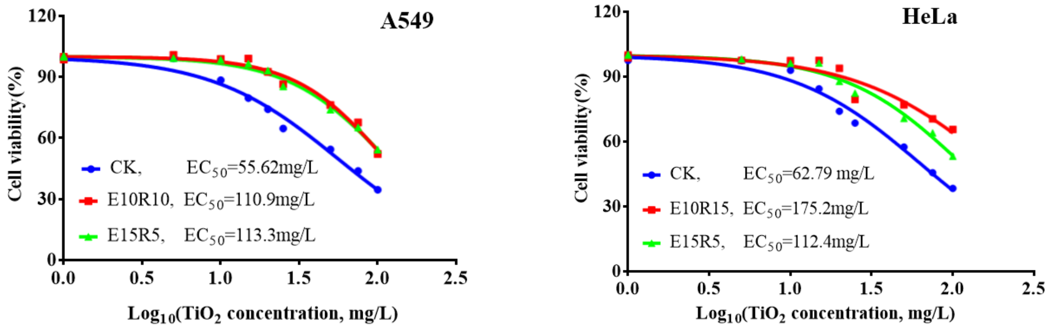

Figure S8 Cell viability of A549 and HeLa cells at 20th passage induced by 24 h exposure to nano-TiO_2.
